# Supplementary material for: ExoSloNano: multimodal nanogold labels for identification of macromolecules in live cells and cryo-electron tomograms
Source: Nat Methods. 2025 Nov 28;23(1):131–42. doi: 10.1038/s41592-025-02928-4 (PMC12791015; doi:10.1038/s41592-025-02928-4)
Supplement: Supplementary file 1 — Reporting Summary [file 41592_2025_2928_MOESM1_ESM.pdf]

nature portfolio

Corresponding author(s): Elizabeth Villa
NMETH-A58398

Last updated by author(s): 09/10/2025

Reporting Summary

Nature Portfolio wishes to improve the reproducibility of the work that we publish. This form provides structure for consistency and transparency in reporting. For further information on Nature Portfolio policies, see our Editorial Policies and the Editorial Policy Checklist.

Statistics

For all statistical analyses, confirm that the following items are present in the figure legend, table legend, main text, or Methods section.

n/a	Confirmed	
☐	☒	The exact sample size (*n*) for each experimental group/condition, given as a discrete number and unit of measurement
☐	☒	A statement on whether measurements were taken from distinct samples or whether the same sample was measured repeatedly
☐	☒	The statistical test(s) used AND whether they are one- or two-sided<br>*Only common tests should be described solely by name; describe more complex techniques in the Methods section.*
☒	☐	A description of all covariates tested
☐	☒	A description of any assumptions or corrections, such as tests of normality and adjustment for multiple comparisons
☐	☒	A full description of the statistical parameters including central tendency (e.g. means) or other basic estimates (e.g. regression coefficient) AND variation (e.g. standard deviation) or associated estimates of uncertainty (e.g. confidence intervals)
☐	☒	For null hypothesis testing, the test statistic (e.g. *F*, *t*, *r*) with confidence intervals, effect sizes, degrees of freedom and *P* value noted<br>*Give P values as exact values whenever suitable.*
☒	☐	For Bayesian analysis, information on the choice of priors and Markov chain Monte Carlo settings
☒	☐	For hierarchical and complex designs, identification of the appropriate level for tests and full reporting of outcomes
☒	☐	Estimates of effect sizes (e.g. Cohen's *d*, Pearson's *r*), indicating how they were calculated

Our web collection on statistics for biologists contains articles on many of the points above.

Software and code

Policy information about availability of computer code

Data collection	SerialEM 4.1.0-beta9, PACEtomo-v1.4.3, MAPS v3.23, AutoTEM v2.4, BD Biosciences LSR-II, BD FACSDiva 8.0.2, Nikon Elements 5.02.02 software, Molecular Devices SpectraMax i3x, Aveole Leonardo
Data analysis	Warp1.09, Warp1.10, RELION-3.1.4, RELION-4.0, IMOD 4.11, Isonet/0.2.1, AreTomo2, ChimeraX (UCSF) w/ ArtiaX v1.7, Prism v 10.3.1, Dynamo v11509, cryolo v1.8.4, DeepFinder, MATLAB version 2019b, ImageJ/FIJI v2.1.0, Imaris v10.2.2, Githubs: (1) ExoSloNano https://github.com/villa-lab/exoslonano , (2) Rosen lab/CATM github https://git.biohpc.swmed.edu/rosen-lab/catm, (3) Script to average neighboring slices of a tomogram: https://github.com/dgvjay/EM_Scripts/average_neighboringSlices.py

For manuscripts utilizing custom algorithms or software that are central to the research but not yet described in published literature, software must be made available to editors and reviewers. We strongly encourage code deposition in a community repository (e.g. GitHub). See the Nature Portfolio guidelines for submitting code & software for further information.

Data

Policy information about availability of data

All manuscripts must include a data availability statement. This statement should provide the following information, where applicable:

- Accession codes, unique identifiers, or web links for publicly available datasets
- A description of any restrictions on data availability
- For clinical datasets or third party data, please ensure that the statement adheres to our policy

Subtomogram averages have been deposited in EMDB (71205, 71211, 71113, 71202). Code and scripts available from the Villa lab and Rosen lab github repositories. Cell lines are available upon request to the corresponding author(s).

Human research participants

Policy information about studies involving human research participants and Sex and Gender in Research.

Reporting on sex and gender	Not applicable
Population characteristics	Not applicable
Recruitment	Not applicable
Ethics oversight	Not applicable

Note that full information on the approval of the study protocol must also be provided in the manuscript.

Field-specific reporting

Please select the one below that is the best fit for your research. If you are not sure, read the appropriate sections before making your selection.

☒ Life sciences ☐ Behavioural & social sciences ☐ Ecological, evolutionary & environmental sciences

For a reference copy of the document with all sections, see nature.com/documents/nr-reporting-summary-flat.pdf

Life sciences study design

All studies must disclose on these points even when the disclosure is negative.

Sample size	For protein abundance experiments (Flow Cytometry experiments), the sample size for each experiment following the established protocol of capturing 10,000 events for each experiment.<br>For room tempature and cryo-ET, sample size is limited to field of of view and available collection area.
Data exclusions	Poorly aligned tilt series were excluded from analysis, as well as micrographs with a poor CTF-fit.
Replication	Quantification of 1.4nm-HAN-594 enhancement in HEK cells, HEK RPL29-Halo cells with and without treatment was analyzed from nine cells for each indicated condition. Quantification of cellular growth following SLO treatment was performed in triplicate. Quantification of cellular volume from HEK RPL29 cells was determined from nine cells. Quantification of ribosomal abundance from HEK RPL29-Halo cells was performed from three independent experiments with two technical replicates. The quantification of mH2A from RPE1 Halo-mH2A cells was performed from four independent experiments. The internalization of 1.4 nm HAN-594 into RPE1 Halo-mH2A was performed from three independent experiments with two technical replicates. Line profile analysis of DAPI and mH2A-Halo intensity was analyzed from five nuclei. Chromatin intensity from ChromEMT experiments were analyzed from three tomograms. CryoFIB/ET was performed independently three times with reproducible results.
Randomization	Half-sets during subtomogram averaging were assigned randomly automatically by RELION-v3.1/RELION-v4 during 3D-auto refinements. Other experiments were not subject to randomization.
Blinding	Investigators were not blinded during data collection.

Reporting for specific materials, systems and methods

We require information from authors about some types of materials, experimental systems and methods used in many studies. Here, indicate whether each material, system or method listed is relevant to your study. If you are not sure if a list item applies to your research, read the appropriate section before selecting a response.

Materials & experimental systems

n/a	Involved in the study
☐	☒ Antibodies
☐	☒ Eukaryotic cell lines
☒	☐ Palaeontology and archaeology
☒	☐ Animals and other organisms
☒	☐ Clinical data
☒	☐ Dual use research of concern

Methods

n/a	Involved in the study
☒	☐ ChIP-seq
☐	☒ Flow cytometry
☒	☐ MRI-based neuroimaging

Antibodies

Antibodies used	Anti-HaloTag® Monoclonal Antibody (Promega #G921A) diluted 1:5000, Anit-MAP2 antibody (Abcam catalog #EPR19691) diluted to 0.5 nM.
Validation	Promega provided validation of this antibody. This antibody was used for western blot analysis to verify expression of the HaloTag in HEK 293T RPL29-Halo cells.

Eukaryotic cell lines

Policy information about cell lines and Sex and Gender in Research

Cell line source(s)	CRISPR knock-in HEK 293T RPL29-Halo was a gift from An et al. (2020), WT HEK 293T was obtained from ATCC (CRL-3216), WT RPE1 was obtained from ATCC (CRL-4000) from which RPE1 Halo-mH2A was generated in this study. CRISPR knock-in U2OS CTCF-Halo was a gift from Cattoglio et al. (2019).
Authentication	CRISPR knock-in HEK 293T L29-Halo were validated through Sanger sequencing of the endogenous RPL29 gene. The gene product was verified by a Western blot against HaloTag, and fluorescence microscopy. CRISPR knock-in RPE1 was validated through Sanger sequencing.
Mycoplasma contamination	Cell lines were not tested for mycoplasma.
Commonly misidentified lines (See ICLAC register)	WT HEK 293T was obtained from ATCC (CRL-3216), WT RPE1 was obtained from ATCC (CRL-4000) and from which RPE1-Halo-mHA was generated.

Flow Cytometry

Plots

Confirm that:

☒ The axis labels state the marker and fluorochrome used (e.g. CD4-FITC).

☒ The axis scales are clearly visible. Include numbers along axes only for bottom left plot of group (a 'group' is an analysis of identical markers).

☒ All plots are contour plots with outliers or pseudocolor plots.

☒ A numerical value for number of cells or percentage (with statistics) is provided.

Methodology

Sample preparation	The absolute protein abundance of H2AFY was determined through flow cytometry when compared to a known standard, U2OS C32 Halo-CTCF cells which were cultured in DMEM supplemented with 1 g/L Glucose and 110 mg/L sodium pyruvate (Gibco, 10567-014) [44]. U2OS C32 HaloTag-CTCF cells were a gift from Robert Tijan's lab (UC Berkeley). U2OS C32 Halo-CTCF cells, HEK293T RPL29-Halo cells, and RPE1 H2AFY-Halo cells were cultured in 6 well plates to 90% confluency, to which 1 uM HaloTag TMR ligand (Promega, G825A) was supplied for 30 minutes. Cells were washed twice with 1x DPBS, trypsinized with 0.5% Trypsin (Gibco 15400054), gently resuspended in HBSS (Gibco 14025134), spun down and gently resuspended in HBSS with 50 ug/ml deoxyribonuclease I from bovine pancreas (Sigma D4263). For each sample, >10,000 events/rates were analyzed on a BD Biosciences LSR-II flow cytometer using a 561 nm excitation laser line and a 582 / 15 band pass filter cube. Absolute protein abundance was determined using an established protocol, Cattoglio et al. (2019).
Instrument	BD Biosciences LSR-II
Software	BD FACSDiva 8.0.2
Cell population abundance	>10,000 TMR positive events

Gating strategy	Gating was performed in order to isolate single cells based on fluorescence for TMR.

☒ Tick this box to confirm that a figure exemplifying the gating strategy is provided in the Supplementary Information.
