# Supplementary material for: ExoSloNano: multimodal nanogold labels for identification of macromolecules in live cells and cryo-electron tomograms
Source: Nat Methods. 2025 Nov 28;23(1):131–42. doi: 10.1038/s41592-025-02928-4 (PMC12791015; doi:10.1038/s41592-025-02928-4)
Supplement: Supplementary file 3 — Source Data Table 1: Raw table of the number of nanogold particles found in the indicated conditions from Fig. 1h (N = 9 cells). [file 41592_2025_2928_MOESM3_ESM.docx]

**Source Data Table 1**: **Raw table of the number of nanogold particles found in the indicated conditions from figure 1h (N = 9 cells)**

| **Number of particles visualized in HEK 293T RPL29-Halo cells treated with 1.4 nm-HAN-488** | **Number of particles visualized in HEK 293T RPL29-Halo cells treated without 1.4 nm-HAN-488** | **Number of particles**  **visualized in WT HEK 293T cells treated with 1.4 nm-HAN-488** |
| --- | --- | --- |
| 5863 | 462 | 390 |
| 5832 | 350 | 349 |
| 5206 | 297 | 273 |
| 5716 | 124 | 432 |
| 5858 | 151 | 259 |
| 3518 | 134 | 577 |
| 3732 | 99 | 442 |
| 5501 | 497 | 447 |
| 2847 | 634 | 518 |
