# Supplementary figures and images for: ExoSloNano: multimodal nanogold labels for identification of macromolecules in live cells and cryo-electron tomograms
Source: Nat Methods. 2025 Nov 28;23(1):131–42. doi: 10.1038/s41592-025-02928-4 (PMC12791015; doi:10.1038/s41592-025-02928-4)

BD FACSDiva 8.0.2

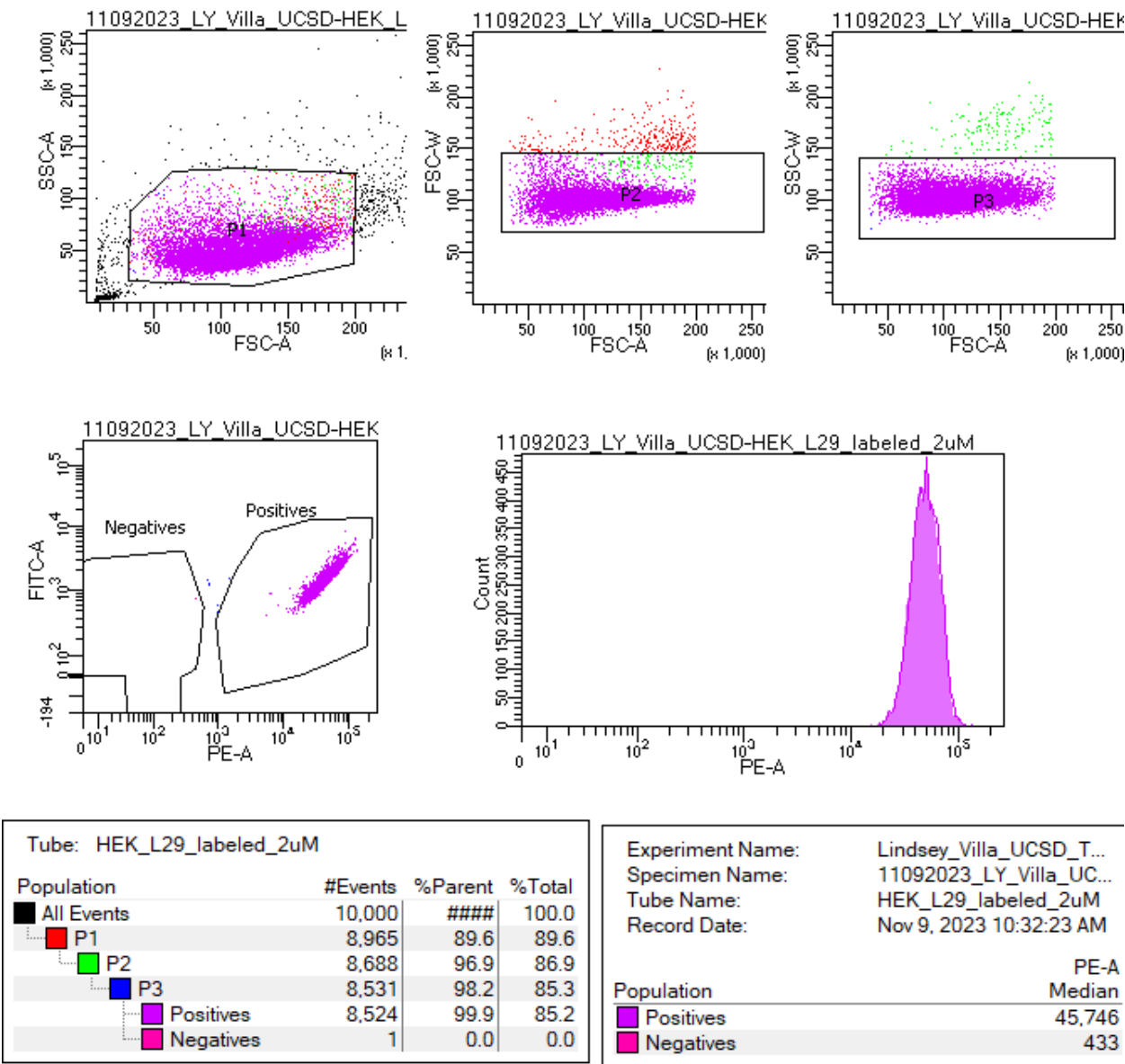

Supplement: Supplementary file 4 — Example gating strategy for quantifying protein abundance by flow cytometry. [file 41592_2025_2928_MOESM4_ESM.pdf]

Source Figure 3

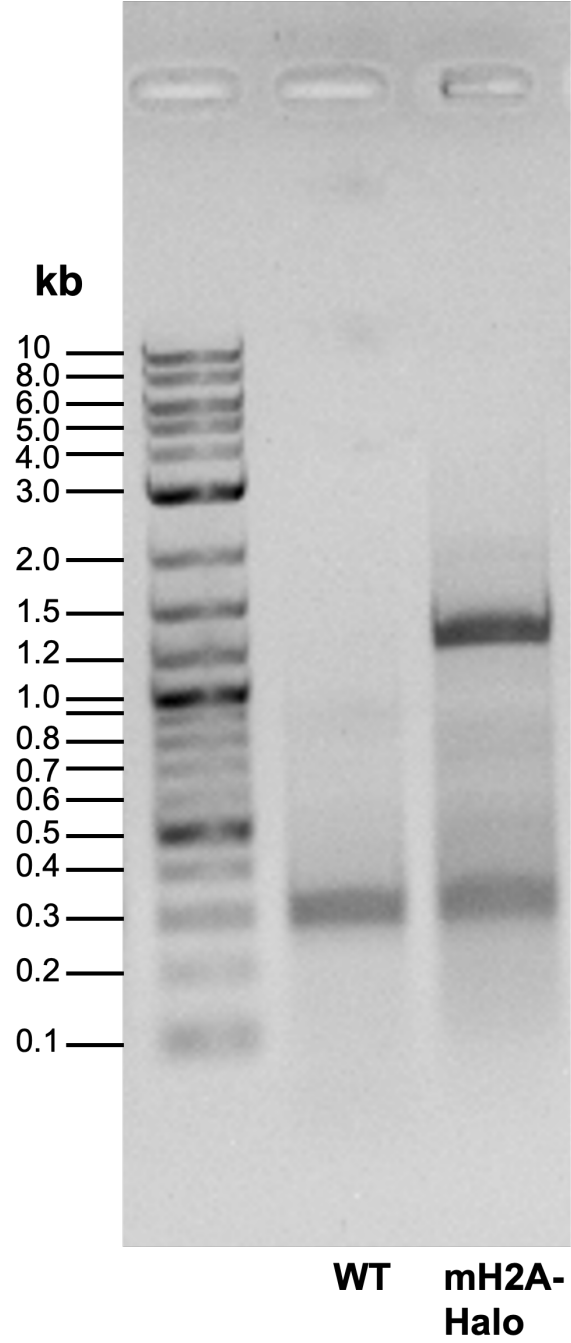

Supplement: Supplementary file 5 — Source data for Fig. 3. [file 41592_2025_2928_MOESM5_ESM.pdf]
